# Standardization of *Juniperus macrocarpa* Sibt. & Sm. and *Juniperus excelsa* M. Bieb. Extracts with Carbohydrate Digestive Enzyme Inhibitory and Antioxidant Activities 

**DOI:** 10.22037/ijpr.2021.114838.15055

**Published:** 2021

**Authors:** Hasya Nazlı Gök, Nilüfer Orhan, Burçin Özüpek, Sultan Pekacar, Şeyma Nur Selvi, Didem Deliorman Orhan

**Affiliations:** *Department of Pharmacognosy, Faculty of Pharmacy, Gazi University, 06330 Etiler, Ankara, Turkey.*

**Keywords:** Antidiabetic, antioxidant, Juniperus macrocarpa, Juniperus excelsa, RP-HPLC

## Abstract

*Juniperus *species growing in Turkey are used for various medicinal purposes in traditional folk medicine. We aimed to evaluate *in-vitro* antidiabetic (α-glucosidase and α-amylase inhibition assays), antiobesity (pancreatic lipase inhibition assay), and antioxidant (ABTS and DPPH radical scavenging activities, ferric reducing activity, metal chelating activity, and phosphomolybdenum assay) activities of the extracts obtained from branches, leaves, and fruits of *Juniperus macrocarpa* and *Juniperus excelsa*. The branch (IC_50_ = 67.1 ± 1.7 µg/mL) and leaf ethyl acetate extracts (IC_50_ = 83.4 ± 0.8 µg/mL) of *J. macrocarpa* exhibited the strongest activity on the α-glucosidase enzyme. Besides that, *J. excelsa* leaf methanol extract exerted remarkable α-amylase inhibitory activity (IC_50_ = 950.1 ± 3.5 µg/mL). Only* J. macrocarpa* branch and* J. excelsa *leaf ethyl acetate extract slightly inhibited pancreatic lipase enzyme with 2963.3 ± 736.4 and 2343 ± 557.8 µg/mL IC_50_ values, respectively. The RP-HPLC-DAD analysis results demonstrated that the more active *J. macrocarpa* extracts are richer in agathisflavone, amentoflavone, and umbelliferone than *J. excelsa* extracts. With this study, it is concluded that *J. macrocarpa* branch and leaf ethyl acetate extracts may be a new source of α-glucosidase enzyme inhibitory activity and agathisflavone, amentoflavone can be used in the standardization of the extracts.

## Introduction

Diabetes mellitus is a very serious health problem that leads to long-continued effects for persons and communities. The greatness of the problem has increased conspicuously in the last thirty years, and it is estimated that it would reach approximately 439 million among adult patients by 2030 (1). Type 2 diabetes is closely related to obesity. Obesity is associated with insulin resistance and type 2 diabetes. Preventing weight gain and reducing obesity is a way that slows down the rate of type 2 diabetes (2). For this reason, new drugs of natural or synthetic origin that can both decrease blood sugar levels and reduce oxidative stress caused by diabetes and additionally play a role in weight control are needed in the fight against a common metabolic disorder, diabetes. Plants utilized for the treatment of diabetes traditionally in different geographies are the subject of inspiring studies for many researchers to discover new drug molecules.

Cupressaceae, which include 30 genera and 140 species, belongs to Gymnospermae. The family is represented by the genera *Cupressus* and *Juniperus,* which are large forests in Turkey (3). *Juniperus* L. is a genus of evergreen trees or shrubs and the second most diversified of the conifers, with some 67 species in the world take place at sea level to over the tree line. Junipers are long-lived trees, sometimes living up to 2000 years (4). Today, the fruits, branches, leaves, and essential oils of *Juniperus* species are used for various purposes in cosmetics and folk medicine (3).

In the light of our literature studies, it has been observed that there are many phytochemicals and activity studies related to *Juniperus* species spread in the northern hemisphere, which are represented by about 67 species in the world. As a result of the phytochemical analysis studies carried out on the branches, leaves, fruits, molasses, and essential oils of the *Juniperus* species, the plant contains phenolic acids, coumarins, lignans, sterols, tannins, and terpenes (5, 6). To date, antifertility, analgesic, anti-inflammatory, antimicrobial, antioxidant, antitumor, neuroprotective, hepatoprotective, and hypoglycemic activity studies conducted on genus have supported the ethnobotanical uses of the plant. 

8 taxa belonging to the genus *Juniperus* are listed in Illustrated Flora of Turkey (7). According to the results of folk medicine field studies conducted in Turkey, it has been observed that *Juniperus* *communis, J. oxycedrus, J. foetidissima,* and *J.* *sabina*, which are used for a long time in the treatment of several diseases, are also used in lowering blood sugar. *In-vitro* antidiabetic activity potentials of* J. communis* var. *saxatilis, J. drupacea, J. foetidissima, J. oxycedrus* ssp. *oxycedrus* and *J.* *sabina *have been evaluated in our previous studies (8-10). The results of our studies have proven that juniper berries, branches, and leaves have promising activity against diabetes mellitus, consistent with their folkloric use. So that, other *Juniperus* species should be evaluated in further studies in terms of their antidiabetic activities. 

In this study, *J. macrocarpa* and *J. excelsa *species, whose *in-vitro* antidiabetic, antiobesity, and antioxidant activity potentials have not been studied so far, were evaluated for these activities. For this purpose, antidiabetic (α-amylase and α-glucosidase inhibitory) and antiobesity (pancreatic lipase inhibitory) activities of water, methanol, and ethyl acetate extracts prepared from branches, fruits, and leaves of two *Juniperus* species were assessed. Antioxidant activities of the extracts were determined by phosphomolybdenum assay, metal chelating activity, ferric reducing antioxidant power, 2,2-diphenyl-1-picrylhydrazyl (DPPH), and 2,2′-azino-bis(3-ethylbenzothiazoline-6-sulfonate) radical cation (ABTS^•+^) scavenging activity assays. While the total phenolic and flavonoid contents of the extracts were measured using the Folin-Ciocalteu method, the RP-HPLC method was used to standardize the extracts.

## Experimental

*Plant materials*

*J. macrocarpa* Sibt. & Sm. (Cupressaceae) was collected near Alaçatı-İzmir, Turkey, in June 2017. The voucher specimens have been stored in the Gazi University Faculty of Pharmacy Herbarium under the herbarium code of GUE 3476, 3477. *J. excelsa *M. Bieb. was collected from Kahramanmaraş-Göksun near Kavsut, Turkey in August 2017. Voucher specimens have been stored in the same herbarium with the code GUE 3478. 

*Preparation of the plant extracts*

Water extract: Dried and powdered plant materials (branches, fruits, and leaves, 5 gr) were extracted with hot water (4% w/v) on a heating-magnetic stirrer for 6 h. Extracts were filtered from filter paper, and the residues were extracted again with the same procedure. Filtered water extracts were combined and freeze-dried. 

Ethyl acetate (EA) and methanol (MeOH) extracts: Dried, and powdered plant materials (5 g for each solvent) were extracted separately with ethyl acetate and methanol (2.5% w/v) on a shaker for 18 h at room temperature. Then extracts were filtered from filter paper, and this procedure was repeated two more times. The extracts were dried by using a rotary evaporator. Yields (w/w) of the extracts were calculated and given in Table 1.

*Determination of total phenolic content*

For the measuring of total phenolic contents of the extracts, Folin-Ciocalteu reagent (10% w/v) was added to the extract. After 5 min of incubation, sodium carbonate solution was added to the wells. The absorbance of the mixtures was read by a microtiter plate reader (Versa Max, Molecular Devices, USA) at 735 nm after the incubation for 30 min in a dark place at room temperature. The total phenolic content was given as mg of gallic acid equivalents (GAE)/g extract. The equation of calibration curve equation was; y (Abs.) = 5.7122 x (Conc.) + 0.0221 and the determination coefficient was r^2^ = 0.9970 (11). 

*Determination of total flavonoid content*

For the measuring of total flavonoid contents of the extracts, aluminum chloride (10%) and sodium acetate (1M) solutions were added to the extracts. After 30 min at 25 °C, the absorbance of the mixture was measured by a microtiter plate reader at 415 nm. Results were given as mg of quercetin equivalents (QE)/g extracts (12). The equation of calibration curve was; y (Abs) = 7.259 x (Conc.) - 0.0555 and the determination coefficient was r^2 ^= 0.9998.

*Enzyme assays*

*α-Glucosidase inhibitory activity assay*

The extracts were preincubated with α-Glucosidase enzyme solution (type IV) in phosphate buffer solution (0.5 M, pH 6.5) for 15 min at 37 °C on a 96-well plate. Then, p-nitrophenyl-α-D-glucopyranoside solution (PNG, 20 mM, Sigma) was added to the wells. The microtiter plate was incubated at 37 °C for 35 min. Acarbose (Bayer, Turkey) was the reference compound. The elevation of the absorption at 405 nm due to the formation of p-nitrophenol was measured by a microtiter plate reader (9). The α-glucosidase inhibitory activity results were given as IC_50_ values (Table 2).

*α-Amylase inhibitory activity assay*

 The plant extract was mixed with the α-Amylase (EC 3.2.1.1, Sigma, type VI) enzyme solution, the mixtures were incubated at 37 °C for 5 min, and substrate solution (0.5% potato starch prepared in phosphate buffer (pH 6.9)) was added. After 3 min incubation at 37 °C, 3,5-dinitrosalicylic acid (DNS) color reagent (96 mM DNS, 5.31 M sodium potassium tartrate in 2 M NaOH) was added to the mixtures, and the tubes were put into an 85 °C heater. After 15 min, the tubes were cooled immediately on ice. Absorbances of the mixtures were read by a microtiter plate reader at 540 nm. Acarbose was used as a reference. The standard maltose calibration graph was prepared (9). The amount of maltose generated was determined by using the standard maltose calibration graph (y = 0.7785 x + 0.0089 and r^2 ^= 0.9925) and the obtained net absorbance. The α-amylase inhibitory activity results were given as IC_50_ values (Table 2).

*Pancreatic lipase inhibitory activity assay*

The pancreatic lipase inhibitory activity of the extracts was determined using our previously published method (13). Pancreatic lipase enzyme type II solution (Sigma Co., St. Louis, USA) was prepared in 4-morpholinepropanesulfonic acid (10 mM) and ethylenediaminetetraacetic acid (EDTA, 1 mM) buffer solution pH 6.8. Extracts were dissolved in ethanol solution (80% w/v) at logarithmic concentrations. The extracts were preincubated with enzyme solution in Tris buffer (pH 7.0, containing Tris–HCl, 100 mM, and CaCl_2_, 5 mM) in a 96-well plate for 15 min at 37 °C. Then, 4-nitrophenyl butyrate (Sigma) was added to the wells. The microtiter plate was incubated at 37 °C for 30 min. The elevation of the absorption at 405 nm due to the formation of p-nitrophenol was measured by a microtiter plate reader. Orlistat (Roche) was used as a reference. The pancreatic lipase inhibitory activity results were given as IC_50_ values (Table 2).

*Antioxidant activity assays*

*Metal chelating capacity*

For the determination of the metal-chelating effect of the samples on the Fe^+2^, samples were incubated with ferrozine (5 mM) and FeCl_2_ solution (2 mM) for 10 min, the absorbances were read at 562 nm by a microtiter plate reader. The inhibiting effect of formation of ferrozine-Fe^+2^ complex was determined using this formula: Activity % = [(A_Control_– A_Sample_)/A_Control_] × 100. EDTA (ethylenediaminetetraacetic acid) was the reference compound. Metal chelating capacity results were given in Table 3. 

*Ferric-reducing antioxidant power*

The extracts were mixed with K_3_Fe(CN)_6_ solutions in a phosphate buffer (0.1 mol/l, pH 7.2), then were incubated for 60 min at 37°C. After that, trichloroacetic acid and FeCl_3_ solutions were added. The absorbance of the samples and the reference compound ascorbic acid were measured at 700 nm by a microtiter plate reader (Table 3) (9).

*Total antioxidant capacity (TAC) by phosphomolybdenum assay*

Molybdate reagent solution was added to the extracts, and tubes were vortexed. After the incubation at 90 °C for 90 min, the tubes were cooled in an ice bath. The absorbances of the extracts were measured by a microtiter plate reader at 695 nm, and the results were given as mg ascorbic acid equivalent (AAE)/g extract (9). Calibration curve equation was; y = 0.0473x + 0.03787 and the determination coefficient was r^2^ = 0.9995. Quercetin was used as a reference compound. Total antioxidant capacity results were given in Table 3.

*ABTS radical scavenging activity*

ABTS·^+^ radical cation scavenging assay was generated by using a spectrophotometric method that was described in Orhan *et al. *(9). Potassium persulphate solution (2.45 mM) was mixed with ABTS (7 mM). The mixture was incubated for 16 hours in the dark at 20 °C. The pH-adjusted ABTS solution with phosphate buffer solution was added to the extracts. After the vortex, the absorbances of the samples were read at 734 nm. by a microtiter plate reader. Gallic acid was used as a reference compound. ABTS radical scavenging activity (inhibition %) = [(A_Control_– A_Sample_)/A_Control_] × 100 (Table 4).

*DPPH radical scavenging activity*

DPPH solution was added to the extracts in a 96 well-plate and incubated in the dark for 30 min. Then, the absorbance of the extracts and reference compound was measured at 520 nm by a microtiter plate reader. As the reference compound was used Ascorbic acid. DPPH radical scavenging activity (inhibition%) = [(A_Control_– A_Sample_)/A_Control_] × 100 (Table 4).

*Standardization of the extracts by using the RP-HPLC method*

Qualitative and quantitative analyzes of amentoflavone, agathisflavone, and umbelliferone in the extracts were performed using the RP-HPLC method. Amentoflavone and agathisflavone were isolated from *Rhus coriaria* L. leaves (16). Umbelliferone was provided by Sigma-Aldrich company (Cas No: 93-35-6). For the analysis, HP Agilent 1260 series LC System and ACE 5 C18 (5 μm, 150 mm × 4.6 mm) column were used. The gradient system was started from the mobile phase contained 10% solvent A (acetonitrile:water: formic acid, 50:50:0.5) and 90% solvent B (water: formic acid, 100:0.5) to 100% solvent A for the 30 min. The flow rate was 1 mL/min, and the injection volume was 20 µL. Detection was carried out at a wavelength of 340 nm by a UV detector. 

*Validation*

The quantitative analysis was conducted with the external standard method. Standard solutions of amentoflavone, agathisflavone, and umbelliferone were prepared with 5 different concentrations. To create the calibration curve, standard substances were analyzed 3 times in HPLC, and the average of the areas under the peak was calculated for each concentration. The extracts were prepared at 5 mg/mL concentration. Each solution was filtered with 0.45 µM membrane filters before injection. The identification of the validation parameters was based on the International Conference on Harmonization (ICH) validation and analytical procedures Q2 (17). Based on the procedure limit of detection (LOD), recovery, limit of quantitation (LOQ), and precision parameters were determined (Tables 5 and 6).

*Statistical Analysis*

All analyses were performed in triplicates. All values are given as the mean ± standard deviation (SD). Calculations and linear regression analyses were carried out by using GraphPad InStat and Microsoft Excel software. A difference in the values of *p* < 0.05 was evaluated to be statistically significant (^*^p < 0.05, ^**^p < 0.01, ^***^p < 0.001). The correlation coefficient was calculated using Microsoft Excel 2016.

## Results

*Determination of total phenolic and flavo-noid content*

The highest extract yield was determined in *J. excelsa* fruit methanol extract (59.43% w/w), and the lowest extract yield was found in *J. macrocarpa* branch ethyl acetate extract (4.38% w/w). Total phenolic contents of the extracts were given as mg GAE/g extract. For this purpose, a calibration graph was made from different dilutions of gallic acid, and the calculations were made according to this graph. Considering all studied plant parts, it has been observed that the total phenolic contents of *J. macrocarpa *extracts are much higher than those of *J. excelsa* extracts (except branch water and methanol extracts). It was determined that the total phenolic contents of *J. macrocarpa* extracts ranged between 17.90 and 232.58 mg GAE/g extract, and the branch extracts of the plant had the highest total phenolic content. In general, the phenolic content of the fruits is low compared to other parts for both species.

Total flavonoid contents of the extracts were given as mg QE/g extract. The total flavonoid content of both species ranged from 7.83 to 62.64 mg QE/g extract. The results showed that *J. excelsa* extracts had higher flavonoid contents than *J. macrocarpa *extracts. When the total flavonoid contents of the extracts were compared, *J. macrocarpa* fruit water and methanol extracts were found to have the least flavonoid contents. 

*Enzyme inhibitory activity*

*α-Glucosidase inhibitory activity*

Among the extracts tested,* J. macrocarpa* branch (IC_50_ = 67.1 ± 1.7 µg/mL) and leaf (IC_50_ = 83.4 ± 0.8 µg/mL) ethyl acetate extracts displayed the highest inhibitory activity. Acarbose used as reference exhibited α-glucosidase inhibitory activity with an IC_50_ value of 0.4 µg/mL. In other words, the extracts having the highest activity demonstrated moderate inhibitory activity against α-glucosidase compared with acarbose. *J. excelsa* extracts (IC_50_ values, 379.6-2696.6 µg/mL) were extremely weak in inhibiting α-glucosidase enzyme compared to *J. macrocarpa* extracts (IC_50_ values, 67.1-775.1 µg/mL). Meanwhile, ethyl acetate and water extracts of the fruits and water extract of leaves of *J. excelsa* did not show any inhibitory activity on this enzyme.

*α-Amylase inhibitory activity*

None of the *J. macrocarpa* extracts had any inhibitory effect against the α-amylase enzyme, while only *J. excelsa* leaf methanol extract (IC_50_ = 950.1 ± 3.5 µg/mL) showed a weak effect compared to acarbose (IC_50_ = 235.4 ± 29.5 µg/mL).

*Pancreatic lipase inhibitory activity*

Except for the *J. macrocarpa* branch (IC_50_ = 2963.3 ± 736.4 µg/mL) and *J. excelsa* leaf (IC_50_ = 2343.0 ± 557.8 µg/mL) ethyl acetate extracts, all the extracts were unable to inhibit pancreatic lipase enzyme. It was concluded that the inhibitory effect of the extracts was extremely weak when the IC_50_ values of the active extracts were compared to the orlistat (IC_50_ = 83.3 ± 7.3 µg/mL) used as a reference.

*Antioxidant activity*

*Metal chelating capacity*

The results of the metal-chelating activity assay were found to be highly controversial. *J. macrocarpa* water and ethyl acetate extracts were found so potent for metal chelating capacity despite the fact that that methanol extracts did not have any effect. EDTA was used as a reference for this assay and showed an extremely strong effect (>100.00%) at all tested concentrations. On the other hand, only fruit ethyl acetate (96.81%) and leaf methanol (99.31%) extracts of *J. excelsa* at 3 mg/ml concentration exerted high activity on metal chelating (Table 3).

*Ferric-reducing antioxidant power*

In this assay, the activities of the extracts were evaluated by comparing the absorbance values of the extracts to the absorbance value of reference ascorbic acid. In addition to the *J. macrocarpa* branch and leaf ethyl acetate (3.12 ± 0.15 and 3.19 ± 0.06, respectively) and methanol extracts (branch 3.31 ± 0.02 and leaf 3.02 ± 0.07, respectively), *J. excelsa* branch methanol and ethyl acetate extracts (3.59 ± 0.03 and 3.50 ± 0.04, respectively) at 3 mg/ml showed high absorbance values as much as the ascorbic acid (3.59 ± 0.09). Among the tested extracts, fruit extracts of both species were considered to be less effective than the others since their absorbance values were lower (ranging from 0.23 ± 0.01 to 1.52 ± 0.14) (Table 3).

*Total antioxidant capacity (TAC)*

*J. excelsa* leaf methanol extract had the highest TAC value (5867.00 ± 25.22 mg AAE/g extract) compared to reference quercetin (16057.79 ± 702.89 mg AAE/g extract), followed by *J. macrocarpa* fruit ethyl acetate extract (3083.86 ± 4.88 mg AAE/g extract) and *J. excelsa* fruit ethyl acetate extract (3006.34 ± 22.87 mg AAE/g extract). *J. macrocarpa* branch ethyl acetate extract exhibited a poor antioxidant potential with 300.21 ± 8.46 mg AAE/g extract Water extracts of both *Juniperus* species, and methanol extracts of *J. macrocarpa* were found to be ineffective in this assay (Table 3).

*ABTS radical scavenging activity*

*J. excelsa *leaf water extract (>100.00%), branch water (99.81%), and branch methanol (>100.00%) extracts showed the highest ABTS radical scavenging effect among the extracts belonging to both *Juniperus *species. On the other hand*, *all leaf and branch extracts, except *J. excelsa* leaf ethyl acetate, showed an extremely strong ABTS radical scavenging effect with inhibition values ranging from >100.00% to 74.39% at 3 mg/ml concentration while gallic acid used as reference displayed inhibition of 99.42% at the same concentration (Table 4). 

*DPPH radical scavenging activity*

Since many extracts precipitated at a concentration of 3 mg/ml in this experiment, percentage inhibition values of the extracts at 1 mg/ml concentrations were compared to evaluate the DPPH radical scavenging effect. Ascorbic acid used as a reference showed 87.29% radical scavenging activity at 1 mg/mL, while *J. macrocarpa* branch (80.23%) and fruit (84.81%) methanol extracts showed extremely high activity. On the other hand, *J. macrocarpa* branch ethyl acetate (69.69%)* J. excelsa* branch (66.67%), leaf (62.42%), and fruit (61.32%) methanol extracts have been observed to display moderate scavenging activity compared to ascorbic acid (Table 4).

*Standardization of the extracts by using the RP-HPLC method*

RP-HPLC method was applied to perform qualitative and quantitative analysis of two bioflavonoids, amentoflavone and agathisflavone, and coumarin umbelliferone in different parts of both *Juniperus* species. This analysis aimed to identify the marker compound(s) for active extracts. Among the three compounds analyzed, all extracts were found to be rich in amentoflavone except water extracts. It was determined that *J. macrocarpa* leaf methanol (0.400 ± 0.000 g/100 g dry extract) and leaf ethyl acetate extracts (0.767 ± 0.000 g/100 g dry extract) had the highest amentoflavone content. Despite that, all water extracts were lack of both amentoflavone and agathisflavone. When the amount of agathisflavone and umbelliferone in the extracts were evaluated, agathisflavone in *J. macrocarpa* extracts were found to vary between 0.005 ± 0.000 and 0.049 ± 0.000 g/100 g extract, and umbelliferone was ranged from 0.007 ± 0.000 to 0.065 ± 0.003 g/100 g extract. On the other hand, agathisflavone was only determined in leaf ethyl acetate extract of *J. excelsa* with only a small amount (0.019 ± 0.001 g/100 g extract), and umbelliferone was found only in *J. excelsa* leaf water and methanol extracts. Additionally, among all *J. excelsa* extracts, leaf methanol (0.274 ± 0.000 g/100 g extract) and ethyl acetate (0.192 ± 0.005 g/100 g extract) extracts were found to have the highest amentoflavone content (Table 7). 

## Discussion

*J. macrocarpa* and *J. excelsa* species, whose *in-vitro* antidiabetic potentials have not been studied so far, have been investigated in this study. Since diabetes mellitus is a metabolic disorder that causes oxidative stress in the organism, the antioxidant activities of the extracts have also been tested. The findings showed that *J. macrocarpa* branch and leaf ethyl acetate extracts had the highest α-glucosidase enzyme inhibitory activity, while *J. excelsa* extracts were very weak. In parallel with these results, *J. macrocarpa* branch and leaf ethyl acetate extracts showed strong metal chelating, ABTS radical scavenging, and ferric reducing power effects. Also, different extracts of different parts of *J. excelsa* exerted a significant effect in terms of metal chelating, ABTS radical scavenging, and ferric reducing power, at least as much as *J. macrocarpa* extracts. It was seen that the extracts of both *Juniperus *species do not have an inhibitory effect on α-amylase and pancreatic lipase enzymes. When the relationship between total phenolic content and α-glucosidase enzyme inhibitory activities of the extracts was investigated, it was observed that there is a negative correlation (r = -0.49323). Conversely, a moderate and positive correlation (r = 0.47989) was found between the total flavonoid content of the extracts and α-glucosidase enzyme inhibitory activities. 

In a previous study, the antioxidant activity of crude ethanol extract and its ethyl acetate and n-butanol fractions of *J. excelsa *leaves collected from Iran was assessed by using inhibition of lipid peroxidation, DPPH radical scavenging activity, and reducing power. In the study using butylated hydroxytoluene and gallic acid as a reference, it was observed that the ethyl acetate fraction showed higher antioxidant activity compared to other samples. On the other hand, it showed weak antioxidant activity compared to the references (18).

The antioxidant activities of fruit extracts (hexane, methanol, and acetone) of six Turkish *Juniperus *species were performed by DPPH radical scavenging activity and β-carotene bleaching assays. It was reported that the acetone extract of *J. excelsa* is significantly effective in the β-carotene-linoleic acid (19).

Orhan *et al. *(20) examined the antioxidant activities (DPPH and superoxide radical scavenging, metal chelating, and ferric reducing antioxidant power assays) of water and methanol extracts prepared from the fruits and leaves of some *Juniperus *species* (J. communis* ssp. *nana, J. foetidissima, J. excelsa, J. sabina, J. oxycedrus* ssp. *oxycedrus*). They evaluated the activities of the samples and discussed the data by only comparing the results of the extracts with each other. Neither a natural nor a synthetic antioxidant compound were used as a reference in any antioxidant activity assay. As a result, leaf extracts of all tested species were reported to show higher activity.

In a study on samples collected from Iran, the antioxidant activities of *J. excelsa* fruit ethanol extracts and its fractions were evaluated using two different methods (DPPH radical scavenging and reducing power assays). The highest DPPH radical scavenging activity was detected in the *n*-butanol fraction (21).

Taviano *et al. *(22) examined the antioxidant potential of methanol and water extracts of branches of *Juniperus* species (*J. oxycedrus* subsp. *macrocarpa, J. communis* var. *communis, J. drupacea, J. communis* var. *saxatilis, *and* J. oxycedrus* subsp. *oxycedrus*) collected from Turkey. *J. oxycedrus* subsp. *macrocarpa* extracts showed the highest effect in both DPHH scavenging activity and lipid peroxidation inhibitory activity.

In this study, antioxidant activity experiments have been carried out to investigate whether extracts with *in-vitro* antidiabetic effects have the potential to combat oxidative stress caused by diabetes. For this reason, it is not possible to establish a relationship and discuss the previous antioxidant activity results that are related to both *Juniperus* species to our results.

Because *J. communis, J. foetidissima, J. oxycedrus*, and* J. sabina* fruits and leaves are used traditionally against diabetes, the antidiabetic activity of these species was shown *in-vivo* in our previous study (24). The highest antidiabetic and antioxidant effects were found in *J. oxycedrus* ssp. *oxycedrus* leaf and fruit extracts, as a result of the study, the active compounds were isolated by bioactivity guided fractionation study. To clarify the mechanism of the effect that was shown in *in-vivo* studies, the activity of some *Juniperus* species against α-amylase and α-glucosidase, which are enzymes related to carbohydrate mechanism, were investigated *in-vitro*. Studies have shown that fruit extracts of *J. communis, drupacea, foetidissima, oxycedrus, macrocarpa, *and *sabina* have remarkable inhibitory effects, especially on the α-glucosidase. Also, the high free radical scavenging and other antioxidant effects of the extracts revealed that the extracts might have positive effects on the increased oxidative stress that formed because of diabetes (7, 8, 20, 23 and 24). In the light of the results obtained from the literature searches, no studies on carbohydrate-digesting enzymes on both *Juniperus *species have been found. 

El-Achi *et al. *(25) determined that the gallic acid (11.3 mg/g), cinnamic acid (5.45 mg/g) and ellagic acid (3.18 mg/g), quercetin (0.36 mg/g), and hesperetin (0.38 mg/g) were the most abundant phenolic compounds in the fruit ethanol extract of *J. excelsa* by RP-HPLC method. In this study, it was suggested that the antioxidant effect of the extract was associated with total flavonoid and phenolic contents. Sahin Yaglioglu and Eser (26) analyzed the methanol extracts of *J. excelsa* needles and cones for 9 phenolic compounds by HPLC-TOF/MS method. The results of the analysis indicated that catechin (326.85 ± 20.2 mg/g dry extract) is abundant in the cone extract of the plant, additionally, both extracts contain methyl robustone and sennidin A. Lesjak *et al. *(2017) evaluated the presence of 44 phenolic acids in hydroalcoholic (80%) extracts of *J. excelsa* leaf and fruit by LC-MS/MS analysis. It was observed that the amount of catechin, epicatechin, rutin, apigenin, amentoflavone, and quercitrin in both of the extracts was considerable (27). 

When we investigated the HLPC and LC-MS analysis studies on *J. macrocarpa*, it was seen that there were not many studies. Taviano *et al. *(28) detected the amounts of gallic acid, tyrosol, protocatechuic acid, rutin, apigenin, amentoflavone, cupressoflavone, and hypolaetin-7 pentoside in the fruit methanol extracts of *J. oxycedrus* L. subsp. *macrocarpa* (Sibth. & Sm.) Ball. by HPLC-DAD-ESI-MS analysis. Cupressoflavone from flavonoids, protocatechuic acid from phenolic acids was found to be higher than other compounds. Moreover, by LC-MS/MS analysis, it was determined by Lesjak, *et al. *(27) that catechin, rutin, epicatechin, quercitrin, and amentoflavone were dominant phenolic compounds in hydroalcoholic (80% methanol) extracts of the leaves and cones of *J. macrocarpa* samples collected from Serbia. 

For the first time in this study, the presence of agathisflavone in *J. macrocarpa* and *J. excelsa* extracts (branch, leaf, and fruit) were determined by RP-HPLC analysis. In two studies (7, 8) that were previously conducted on different species, amentoflavone and umbelliferone were thought to contribute to antidiabetic and antioxidant effects, and their quantities were determined. Besides, they were detected in both *Juniperus *species in this study. Results of our analysis indicated that more active *J. macrocarpa* extracts are richer in agathisflavone, amentoflavone, and umbelliferone compared to *J. excelsa* extracts. Also, unlike other publications that we refer to in this study, for the first time, the phytochemical contents of three different extracts of three different parts of these species were examined comparatively.

As a result, *J. macrocarpa* branch ethyl acetate extract was found to have potent α-glucosidase enzyme inhibitor activity. Likewise, this extract has strong metal chelating, ferric reducing power, and ABTS radical scavenging activity, it is thought to be effective in oxidative stress due to diabetes. Additionally, it can also be said that amentoflavone and agathisflavone can be used in the standardization of this extract. Since there is a moderate relationship between the total flavonoid content of the extracts and the inhibitory effects of the α-glucosidase enzyme, further studies have to be planned to investigate the *in-vivo* antidiabetic effects of *J. macrocarpa* extracts and to isolate the potential flavonoid compounds responsible for the activity.

**Table 1:** Yield percentages (w/w %), total phenolic and flavonoid contents of *Juniperus *species

	**Plant Part**	**Extract**	**Yield%** **(w/w)**	**Total Phenolic Content** ^a^ **(Mean ± SD)**	**Total Flavonoid Content** ^b^ **(Mean ± SD)**
*J. macrocarpa*	Branch	Water	11.53	92.18 ± 8.44	12.56 ± 0.16
		MeOH	12.55	107.82 ± 3.34	9.99 ± 0.24
		EA	4.38	232.58 ± 8.30	17.33 ± 0.64
	Fruit	Water	23.76	17.90 ± 4.81	7.83 ± 0.29
		MeOH	28.29	44.51 ± 7.64	9.25 ± 0.08
		EA	6.12	19.71 ± 4.60	30.24 ± 3.39
	Leaf	Water	18.14	93.58 ± 7.26	11.37 ± 0.14
		MeOH	26.34	104.44 ± 7.56	21.47 ± 0.35
		EA	6.49	82.79 ± 7.87	45.16 ± 3.96
*J. excelsa*	Branch	Water	9.01	136.30 ± 12.71	34.05 ± 2.31
		MeOH	11.16	137.87 ± 2.98	36.26 ± 0.96
		EA	8.44	77.42 ± 5.10	42.06 ± 2.04
	Fruit	Water	28.48	8.27 ± 1.57	29.22 ± 0.63
		MeOH	59.43	34.47 ± 3.04	31.71 ± 0.63
		EA	12.95	6.58 ± 2.19	50.76 ± 2.50
	Leaf	Water	18.42	102.86 ± 8.58	39.30 ± 0.48
		MeOH	27.95	29.86 ± 2.19	62.64 ± 2.59
		EA	10.95	19.59 ± 4.20	51.18 ± 0.86

^ a^mg GAE/g extract, ^b^mg QE/g extract.

**Table 2:** Enzyme inhibitory activities of *Juniperus* extracts

	**Plant Part**	**Extract**	**IC**_50_ **(µg/mL ± SD**)		
			**α-Glucosidase**	**α-Amylase**	**Pancreatic lipase**
*J. macrocarpa*	Branch	Water	775.1 ± 113.5	-	-
		MeOH	120.4 ± 2.6	-	-
		EA	67.1 ± 1.7	-	2963.3 ± 736.4
	Fruit	Water	-^*^	-	-
		MeOH	290.3 ± 5.0	-	-
		EA	112.1 ± 2.0	-	-
	Leaf	Water	636.5 ± 39.6	-	-
		MeOH	375.3 ± 6.7	-	-
		EA	83.4 ± 0.8	-	-
*J. excelsa*	Branch	Water	1008.5 ± 4.7	-	-
		MeOH	379.6 ± 78.2	-	-
		EA	565.3 ± 33.5	-	-
	Fruit	Water	-	-	-
		MeOH	2696.6 ± 297.4	-	-
		EA	-	-	-
	Leaf	Water	-	-	-
		MeOH	1636.3 ± 68.7	950.1 ± 3.5	-
		EA	2493.0 ± 205.0	-	2343.0 ± 557.8
Reference			0.4 ± 0.2^a^	235.4 ± 29.5^a^	83.3 ± 7.3^b^

^ *^The IC_50_ values are higher 3 mg/mL. ^a^Acarbose, ^b^Orlistat, -: No activity.

**Table 3:** Metal chelating activity, ferric reducing power and total antioxidant capacity of *Juniperus* extracts

	**Plant Part**	**Extract**	**Metal Chelating Activity** **% ± SD**			**Ferric Reducing Power ** **Absorbance ± SD**			**Total Antioxidant Capacity**
			**3 (mg/mL)**	**1 (mg/mL)**	**0.3 (mg/mL)**	**3 (mg/mL)**	**1 (mg/mL)**	**0.3 (mg/mL)**	**1 (mg/mL)**
*J. macrocarpa*	Branch	Water	>100.00^***^	>100.00^***^	83.06 ± 0.95^***^	1.95 ± 0.09	1.09 ± 0.04	0.39 ± 0.01	-
		MeOH	-	-	-	3.31 ± 0.02	1.44 ± 0.06	0.88 ± 0.02	-
		EA	>100.00^**^	>100.00^***^	>100.00^***^	3.12 ± 0.15	2.39 ± 0.08	0.74 ± 0.07	300.21 ± 8.46
	Fruit	Water	> 100.00^***^	85.21 ± 1.87^***^	-	0.59 ± 0.04	0.20 ± 0.03	0.05 ± 0.00	-
		MeOH	-	-	-	1.52 ± 0.14	0.41 ± 0.01	0.12 ± 0.00	-
		EA	70.07 ± 6.95^**^	14.48 ± 3.33^*^	-	0.28 ± 0.01	0.18 ± 0.08	0.04 ± 0.01	3083.86 ± 4.88
	Leaf	Water	>100.00^***^	>100.00^***^	-	2.56 ± 0.13	1.06 ± 0.06	0.34 ± 0.02	-
		MeOH	-	-	-	3.02 ± 0.07	1.20 ± 0.04	0.38 ± 0.02	-
		EA	>100.00^**^	>100.00^***^	99.09 ± 0.01^***^	3.19 ± 0.06	0.68 ± 0.03	0.18 ± 0.01	3422.13 ± 2.22
*J. excelsa*	Branch	Water	-	-	-	2.63 ± 0.05	1.79 ± 0.03	0.58 ± 0.02	-
		MeOH	22.73 ± 6.66^**^	18.14 ± 3.58^*^	13.30 ± 0.96^***^	3.59 ± 0.03	2.03 ± 0.06	0.65 ± 0.01	2301.62 ± 17.98
		EA	-	-	-	3.50 ± 0.04	1.21 ± 0.02	0.43 ± 0.01	2350.00 ± 7.62
	Fruit	Water	-	-	-	0.75 ± 0.01	0.17 ± 0.00	0.06 ± 0.00	-
		MeOH	-	-	-	1.46 ± 0.04	0.57 ± 0.01	0.17 ± 0.00	1885.84 ± 5.59
		EA	96.81 ± 0.14^***^	97.88 ± 0.42^**^	12.21 ± 1.17^***^	0.23 ± 0.01	0.11 ± 0.01	0.04 ± 0.00	3006.34 ± 22.87
	Leaf	Water	-	18.71 ± 4.05^***^	17.66 ± 3.80^***^	2.77 ± 0.04	1.26 ± 0.01	0.37 ± 0.01	-
		MeOH	99.31 ± 0.05^***^	99.72 ± 0.03^**^	18.71 ± 2.98^***^	0.98 ± 0.06	0.31 ± 0.08	0.09 ± 0.00	5867.00 ± 25.22
		EA	-	-	-	0.50 ± 0.04	0.18 ± 0.01	0.06 ± 0.00	1639.18 ± 10.43
Reference			> 100.00^a***^	> 100.00^ a***^	> 100.00^ a***^	3.59 ± 0.09^b^	3.45 ± 0.08^ b^	3.08 ± 0.06^ b^	16057.79 ± 702.89^ c^

-: No activity. SD: Standard Deviation, ^a^EDTA, ^b^Ascorbic acid, ^c^Quercetin, Total antioxidant capacity is given as mg AAE/g extract ± SD, ^*^p < 0.05, ^**^p < 0.01, ^***^p < 0.001.

**Table 4:** DPPH and ABTS radical scavenging activities of *Juniperus* extracts

	**Plant Part**	**Extract**	**DPPH radical scavenging activity** **Inhibition% ± SD**			**ABTS radical scavenging activity** **Inhibition% ± SD**		
			**3 (mg/mL)**	**1 (mg/mL)**	**0.3 (mg/mL)**	**3 (mg/mL)**	**1 (mg/mL)**	**0.3 (mg/mL)**
*J. macrocarpa*	Branch	Water		14.34 ± 3.55^***^	34.96 ± 0.88^***^	95.85 ± 4.81^***^	54.92 ± 4.85^***^	22.78 ± 6.58^***^
		MeOH	70.54 ± 0.27^***^	80.23 ± 0.62^***^	78.91 ± 0.71^***^	96.69 ± 5.54^***^	82.20 ± 0.87^***^	48.31 ± 1.52^***^
		EA	22.48 ± 1.42^*^	69.69 ± 3.36^***^	75.81 ± 0.47^***^	97.25 ± 0.36^***^	96.48 ± 1.55^***^	57.52 ± 0.53^***^
	Fruit	Water		52.95 ± 1.19^***^	26.12 ± 4.37^**^	38.53 ± 4.75^***^	24.12 ± 6.88^***^	9.98 ± 1.96^***^
		MeOH	83.49 ± 0.23^***^	84.81 ± 1.65^***^	70.23 ± 4.03^***^	82.55 ± 5.44^***^	34.03 ± 2.63^***^	17.44 ± 3.28^***^
		EA				63.29 ± 0.63^***^	36.42 ± 0.67^***^	17.72 ± 2.69^***^
	Leaf	Water		31.78 ± 3.58^***^	55.58 ± 4.88^***^	98.52 ± 0.91^***^	68.42 ± 4.33^***^	32.34 ± 3.79^***^
		MeOH		36.51 ± 3.26^***^	30.70 ± 1.99^***^	99.71 ± 0.24^***^	84.88 ± 2.62^***^	44.16 ± 5.15^***^
		EA		49.22 ± 7.61^***^	73.49 ± 0.81^***^	97.04 ± 0.91^***^	77.21 ± 2.28^***^	40.08 ± 0.63^***^
*J. excelsa*	Branch	Water		27.43 ± 1.00^***^	28.46 ± 3.07^***^	99.81 ± 0.19^***^	26.37 ± 0.30^***^	6.64 ± 1.18^***^
		MeOH	63.52 ± 0.27^***^	66.67 ± 0.54^***^	46.70 ± 7.37^***^	>100.00^***^	74.60 ± 0.20^***^	63.64 ± 0.57^***^
		EA	21.54 ± 6.69^***^	58.81 ± 3.57^***^	55.50 ± 1.91^***^	74.85 ± 1.77^***^	18.31 ± 0.97^***^	-
	Fruit	Water	44.03 ± 0.98^***^	36.01 ± 0.72^***^	24.69 ± 2.76^***^	30.17 ± 2.23^***^	-	-
		MeOH	59.43 ± 0.94^***^	61.32 ± 0.82^***^	47.17 ± 0.47^***^	49.26 ± 1.10^***^	-	-
		EA		8.33 ± 5.97^*^	-	13.09 ± 2.31^***^	-	-
	Leaf	Water	51.10 ± 1.19^***^	39.94 ± 1.36^***^	35.38 ± 0.00^***^	>100.00^***^	70.39 ± 0.95^***^	62.86 ± 0.73^***^
		MeOH	23.58 ± 3.86^***^	62.42 ± 1.44^***^	58.49 ± 0.94^***^	74.39 ± 0.47^***^	61.16 ± 0.49^***^	60.19 ± 0.52^***^
		EA		44.65 ± 2.42^***^	37.26 ± 1.70^***^	18.11 ± 1.09^***^	11.04 ± 0.52^***^	-
Reference			87.13 ± 0.48^a***^	87.29 ± 0.71^a***^	87. 98 ± 0.27^a***^	99.42 ± 0.84^b***^	99.03 ± 0.39^b***^	98.32 ± 0.30^b***^

precipitate formation was observed, -: No activity. SD: Standard Deviation, ^a^Ascorbic acid, ^b^Gallic acid, ^*^p < 0.05, ^**^p < 0.01, ^***^p < 0.001.

**Table 5:** Retention times (Rt), linear relationships between peak areas and concentrations, test ranges, LOD and LOQ of *J. macrocarpa*

**Compound **	**Rt **(**min**)	**Standard curve**	**r** ^2^	**Test range** **(ppm)**	**LOD (ppm)**	**LOQ (ppm)**	**RSD%**
**Amentoflavone **	25.821	y = 51.789x - 7.7748	0.9998	0.5-20	0.127	0.386	5.345
**Agathisflavone**	24.853	y = 87.059x - 4.7626	0.9999	0.2-20	0.031	0.094	4.109
**Umbelliferone **	13.730	y = 85.518x - 3.6697	0.9999	0.5-20	0.045	0.138	1.406

**Table 6:** Retention times (Rt), linear relationships between peak areas and concentrations, test ranges, LOD and LOQ of *J. excelsa*

**Compound**	**Rt** **(min)**	**Standard curve**	**r** ^2^	**Test range (ppm)**	**LOD (ppm)**	**LOQ (ppm)**	**RSD%**
**Amentoflavon** **e**	25.374	y = 60.887x+1.0001	0.9999	0.4-50	0.108	0.329	2.650
**Agathisflavon** **e**	24.397	y = 88.329x-2.8497	0.9999	0.4-50	0.064	0.195	0.460
**Umbelliferon** **e**	12.885	y = 84.253x+16.124	0.9999	1-50	0.316	0.958	4.450

**Table 7:** Amentoflavone, agathisflavone and umbelliferone contents (g/100 g dry extract) of branches, fruits and leaf extracts of *Juniperus* species

**Species**	**Plant Part**	**Extract**	**Compound**		
			**Amentoflavone**	**Agathisflavone**	**Umbelliferone**
*J. macrocarpa*	**Branch**	Water	-	-	0.008 ± 0.002
		MeOH	0.123 ± 0.000	0.005 ± 0.000	0.018 ± 0.000
		EA	0.271 ± 0.000	0.012 ± 0.000	-
	**Fruit**	Water	-	-	-
		MeOH	0.071 ± 0.000	0.005 ± 0.000	0.007 ± 0.000
		EA	0.238 ± 0.001	0.013 ± 0.000	-
	**Leaf**	Water	0.023 ± 0.000	-	0.061 ± 0.000
		MeOH	0.400 ± 0.000	0.013 ± 0.000	0.065 ± 0.003
		EA	0.767 ± 0.000	0.049 ± 0.000	0.024 ± 0.001
*J. excelsa*	**Branch**	Water	-	-	-
		MeOH	0.038 ± 0.000	-	-
		EA	0.063 ± 0.000	-	-
	**Fruit**	Water	-	-	-
		MeOH	0.060 ± 0.000	-	-
		EA	0.070 ± 0.001	-	-
	**Leaf**	Water	-	-	0.031 ± 0.008
		MeOH	0.274 ± 0.000	-	0.027 ± 0.003
		EA	0.192 ± 0.005	0.019 ± 0.001	-

-: Not determined.

**Figure 1:**
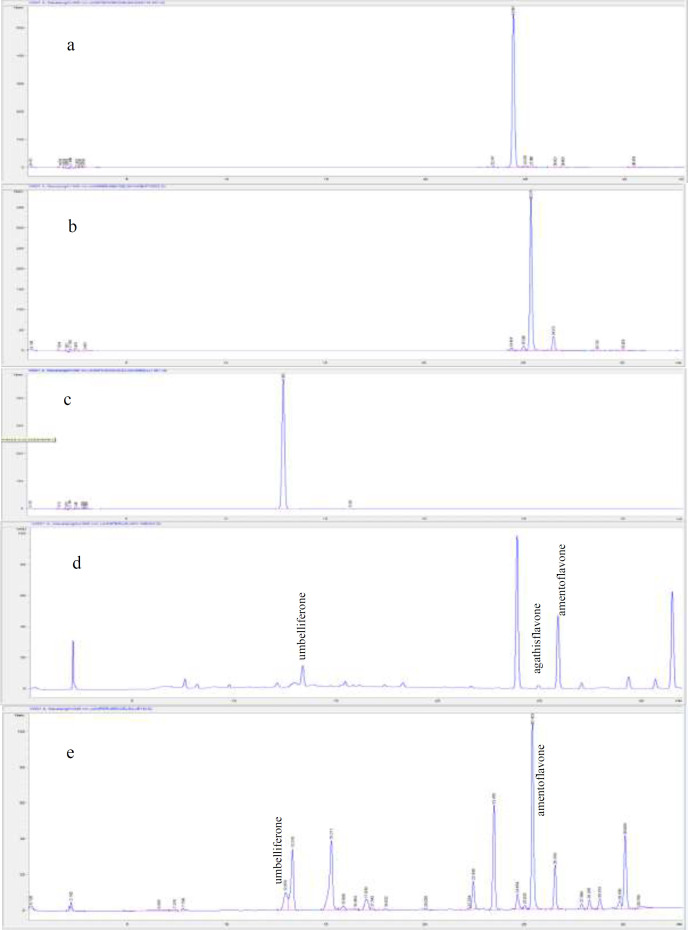
The HPLC chromatograms of standard compounds and the extracts. (a) Agathislavone, (b) Amentoflavone, (c) Umbelliferone, (d) *J. macrocarpa* leaf methanol extract, (e) *J. excelsa* leaf methanol extract)

## Conclusion

Finally, this study was a part of our ongoing studies on the biological activities and phytochemical analysis of* Juniperus *species of Turkey. To sum up, *J. macrocarpa *having a strong α-glucosidase enzyme inhibitory effect had also revealed strong antioxidant activity on different assays. Oxidative stress and elevated free radical formation are known to play an important role in the development of tissue and organ damage in diabetic patients. So that antidiabetic herbal remedies having antioxidant activity as well are so precious in phytotherapy. Amentoflavone, agathisflavone, and umbelliferone were determined as the marker compounds of the active extracts. So that, standardization of the *Juniperus *extracts and preparations could be performed by using these three compounds for the generation of new herbal medicinal products that could be effective in the control of blood glucose concentration. 

## Author contributions

Conceptualization (H. N. Gök, N. Orhan D. Deliorman Orhan), Investigation (H. N. Gök, N. Orhan, B. Özüpek, S.Pekacar, Ş.N.Selvi, D. Deliorman Orhan ), Methodology (H. N. Gök, N. Orhan, B. Özüpek, S.Pekacar, Ş.N.Selvi, D. Deliorman Orhan), Project administration (H. N. Gök, N. Orhan, D. Deliorman Orhan), Resources (H. N. Gök, N. Orhan, B. Özüpek, S.Pekacar, D. Deliorman Orhan), Writing – original draft – (H. N. Gök, N. Orhan, B. Özüpek, S.Pekacar, D. Deliorman Orhan H. N. Gök), Writing – review & editing – (H. N. Gök, N. Orhan, B. Özüpek, S.Pekacar, D. Deliorman Orhan).
